# Comprehensive Assessment of Alfalfa Cultivars for Resistance to *Meloidogyne incognita* Using Multiple Evaluation Indices

**DOI:** 10.3390/life16010093

**Published:** 2026-01-08

**Authors:** Ying Yu, Xu Zhuang, Sobhi F. Lamlom, Dongmei Zhang, Jianli Wang, Linlin Mu, Lijian Xu, Zhongbao Shen, Weibo Han, Jia You

**Affiliations:** 1 College of Advanced Agriculture and Ecological Environment, Heilongjiang University, Harbin 150080, China; yuying12042022@163.com (Y.Y.); sobhifaid@alexu.edu.eg (S.F.L.); xulijian@hlju.edu.cn (L.X.); 2 Institute of Forage and Grassland Sciences, Heilongjiang Academy of Agricultural Sciences, Harbin 150086, China; yangyangcaocao@haas.cn (X.Z.); zhangdongmei@haas.cn (D.Z.); jianli@haas.cn (J.W.); budaoweng@haas.cn (L.M.); shenzhongbao@haas.cn (Z.S.); 3 Plant Production Department, Faculty of Agriculture Saba Basha, Alexandria University, Alexandria 21531, Egypt

**Keywords:** alfalfa, *Meloidogyne incognita*, disease index, egg mass index, resistance screening, root-knot nematode, developmental inhibition, germplasm evaluation

## Abstract

Root-knot nematodes (RKN), especially *Meloidogyne incognita*, threaten global alfalfa crops because of their broad host range and pathogenic nature. Despite its significance, research on resistance is limited. In this study, 24 varieties from China, the US, Canada, Australia, and France were assessed for resistance using the Disease Index (DI) and Egg Mass Index (EMI). Results identified 19 varieties with varying resistance levels and 5 that were susceptible. Chinese Gannong No. 9 was highly resistant (DI: 10) and achieved the highest composite score (91). The US varieties Dryland and Moste were classified as resistant (DI: 14.3% and 12.5%, respectively) and also ranked highly by composite score (65 and 62.5). A moderate correlation between DI and EMI (r = 0.68) led to some inconsistent classifications, including for 2295, Instict, and WL168HQ, highlighting the importance of using multiple complementary metrics for accurate resistance evaluation. Egg mass production was strongly correlated with galling severity (r = 0.70), while root biomass showed no correlation with galling (r = 0.09), indicating root weight is not a reliable resistance indicator. Preliminary infection dynamics showed similar nematode penetration rates at 2 days post-infection across resistant and susceptible varieties. At 7 days post-infection, both resistant and susceptible varieties retained predominantly J2 larvae (78–89%), with no statistically significant differences in developmental stage distributions. These preliminary observations suggest that resistance-associated effects on nematode development, if present, are not strongly expressed at early stages of infection. The mechanistic basis of resistance in alfalfa remains unresolved and warrants further investigation using additional timepoints, histological analyses of feeding-site development, and molecular characterization. Geographically, American varieties displayed broad performance variation, Chinese varieties showed a bimodal distribution, and Canadian varieties exhibited moderate, consistent resistance. These results offer valuable germplasm for breeding and highlight the importance of multiple resistance metrics. Resistant varieties such as Gannong No. 9 provide important genetic resources for developing durable nematode resistance in alfalfa and can guide variety selection in nematode-infested regions.

## 1. Introduction

Alfalfa (*Medicago sativa* L.), also known as lucerne, is a vital crop for global forage systems and has recently been ranked as the third most valuable field crop in the United States by the National Alfalfa and Forage Alliance [1]. Alfalfa is vital for livestock, particularly dairy cattle, due to its excellent nutritional quality (crude protein of 15–22%), high digestibility, and nitrogen-fixing symbiosis with *Sinorhizobium meliloti*, as well as its ability to be harvested multiple times each season [2]. In China, increased demand for high-quality animal protein has led to significant increases in alfalfa consumption, with the country now importing over 1.4 million metric tons of alfalfa hay each year and expanding local cultivation to improve self-sufficiency in forage production [3]. However, the growth and increased intensity of alfalfa production have also raised awareness of nematode-related challenges to productivity and stand longevity.

Plant-parasitic nematodes are some of the most economically significant pests in agriculture, leading to about $157 billion in crop losses globally each year [4]. Root-knot nematodes are recognized as the most damaging group within this category, capable of infecting over 5500 plant species across various taxonomic groups [5,6]. These stationary endoparasites infect host roots during their second juvenile stage (J2), migrate through cortical tissues, and create specialized feeding structures called giant cells within the vascular cylinder [6,7]. The formation of giant cells causes notable changes in the surrounding root tissues, including widespread cellular hypertrophy and hyperplasia, which eventually result in the formation of characteristic root galls [8,9]. These galls hinder water and nutrient absorption, reduce photosynthetic efficiency, and create entry points for secondary pathogens [8,9].

Among the four major RKN species relevant to agriculture, *Meloidogyne incognita* (southern root-knot nematode), *Meloidogyne javanica* (Javanese root-knot nematode), *Meloidogyne arenaria* (peanut root-knot nematode), and *Meloidogyne hapla* (northern root-knot nematode), *Meloidogyne incognita* is the most destructive [5,7]. Its broad host range, global distribution, and high reproductive rate under favorable conditions enhance its threat [10]. Initially confined to tropical and subtropical regions, *Meloidogyne incognita* has expanded into temperate zones due to climate change and the spread of protected cultivation methods like greenhouses and polytunnels [11]. This expansion has enabled it to establish in northern regions previously too cold for survival, making it more common where it was once rare or absent. It reproduces via mitotic parthenogenesis, producing only females, which accelerates population growth even from a single individual [12]. Its short generation time of 3–4 weeks and high egg production of 300–500 eggs per female allow for rapid population growth, often reaching damaging levels within a single crop cycle [13]. Multiple plant-parasitic nematode species pose threats to alfalfa production, including the stem and bulb nematode (*Ditylenchus dipsaci*), root lesion nematodes (*Pratylenchus* spp.), cyst nematodes (*Heterodera* spp.), and root-knot nematodes (*Meloidogyne* spp.) [4,14,15]. Among these, five RKN species have been documented causing economically significant damage to alfalfa: *M. incognita*, *M. hapla*, *M. chitwoodi* (Columbia root-knot nematode), *M. javanica*, and *M. arenaria* [16]. Nematode infestations in alfalfa cause several harmful effects, including stunted shoot growth, chlorosis, wilting under water stress, decreased stand density, shorter stand lifespan, lower forage yield (reductions of 20–60% have been documented in heavily infested fields), reduced forage quality due to lower protein content and digestibility, and increased vulnerability to winter injury and secondary pathogens [17]. The perennial nature of alfalfa cultivation compounds the economic impact, as nematode populations accumulate over the 3–5 year productive life of stands, progressively worsening damage over time [18]. Given the broad host ranges of RKN species and their persistence in soil, infested alfalfa fields also serve as reservoirs that threaten subsequent crops in rotation, including high-value vegetables, particularly in regions where alfalfa is grown in intensive vegetable production systems [19,20]. Despite extensive research on RKN resistance in major crops, knowledge of resistance mechanisms and sources in alfalfa remains limited. As early as 1978, the US developed the Nevada Synthetic YY germplasm, which is resistant to *M. hapla* and *M. incognita*, demonstrating the feasibility of resistance breeding [21]. However, systematic screening of commercial alfalfa for RKN resistance has been sporadic and localized, mainly focusing on *M. hapla* due to its importance in the northern US and Canada [22].

Recent transcriptomic studies in annual crops such as tobacco and cucumber have identified defense-related genes upregulated during *M. incognita* infection [23,24]. However, resistance mechanisms in alfalfa remain largely uncharacterized. Unlike diploid annual model systems, alfalfa is an autotetraploid perennial legume with nitrogen-fixing symbiosis, features that may fundamentally alter resistance pathways. Direct investigation of alfalfa-specific mechanisms through histology, genetics, and molecular biology is needed rather than extrapolation from distant crop relatives. This study aimed to address these gaps through four objectives: (1) screening 24 diverse alfalfa cultivars from different countries to identify resistant germplasm for producers and breeders; (2) comparing DI and EMI to evaluate their correlation and consistency; (3) investigating resistance mechanisms by analyzing differences in nematode infection and larval development between resistant and susceptible varieties to determine if resistance occurs before or after penetration; (4) examining correlations among resistance indicators such as root galling, egg production, and root weight to find reliable markers and understand the relationship between symptoms and nematode success. The findings provide practical benefits for growers and support resistance breeding by identifying resistant germplasm and improving assessment methods, helping develop durable, resistant alfalfa cultivars for sustainable, secure production worldwide.

## 2. Materials and Methods

### 2.1. Plant Materials

**Figure 1:**
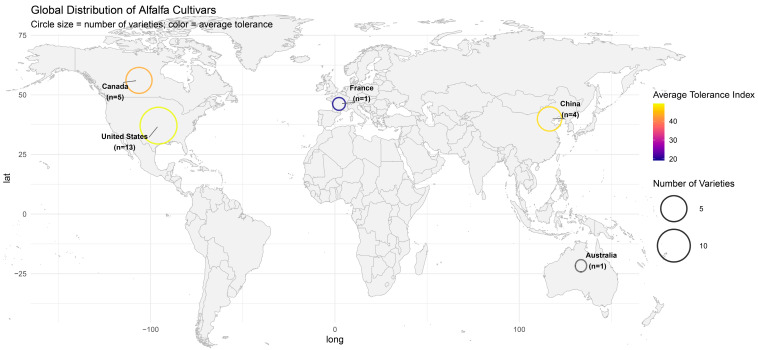
Geographic distribution of tested alfalfa cultivars and their average resistance to *M. incognita*. Circles represent the country of origin for the 24 alfalfa cultivars evaluated in this study. Circle size is proportional to the number of varieties tested from each country (United States: n = 13; Canada: n = 5; China: n = 4; France: n = 1; Australia: n = 1). Color intensity indicates the average composite resistance score for cultivars from each country on a 0–100 scale, where higher values represent greater resistance.

Seeds of 24 alfalfa (*Medicago sativa* L.) cultivars representing diverse geographic origins were obtained from the germplasm collection maintained at the Institute of Forage and Grassland Sciences, Heilongjiang Academy of Agricultural Sciences, Harbin, China (Figure 1). The cultivar panel included four varieties from China (Gannong No. 9, Nongjing No. 1, Aohan, Jiuquan), thirteen from the United States (Kangsai 1, Gibraltar, 218TR, 310SC, Magnum 2, Moste, Dryland, Ordinary, Salt-tolerant Star, Instict, WL343HQ, WL440HQ, WL168HQ), five from Canada (Super Nova, 5020, 2295, 3010, 2065MF), one from France (Catera), and one from Australia (Aurora). These cultivars represent commercially essential materials used in production systems across multiple regions and climate zones.

### 2.2. Nematode Culture and Inoculum Preparation

*M. incognita* populations were initially isolated from heavily infected tomato (*Solanum lycopersicum* L.) roots exhibiting characteristic root galling. The population was maintained and propagated on the highly susceptible tomato cultivar DaHong in a temperature-controlled greenhouse at 22–26 °C to ensure robust nematode multiplication and maintain population vigor. For egg extraction, tomato roots were harvested 35 days after initial infection, when second-generation egg masses were abundant. Roots were thoroughly washed to remove soil and cut into 2–3 cm segments. The root segments were completely submerged in a 0.5% sodium hypochlorite (NaOCl) solution and agitated on a reciprocating shaker at room temperature for 4 min to digest the egg mass’s gelatinous matrices and release eggs. The suspension was immediately passed sequentially through 200-mesh (75 μm) and 500-mesh (25 μm) stainless steel sieves. Eggs retained on the 500-mesh sieve were collected by back-washing into a beaker and examined under a stereomicroscope (Olympus SZX16, Tokyo, Japan) to verify egg quality and viability. Only preparations containing >95% intact, embryonated eggs were used for experiments. For hatching of second-stage juveniles (J2), eggs were transferred onto a nylon mesh (25 μm pore size) supported on a modified Baermann funnel apparatus. A single layer of sterilized tissue paper was placed on the mesh to provide substrate for egg adherence. Sterile distilled water was added to the collection chamber such that the water level remained just above the mesh surface, maintaining constant moisture without submerging eggs. The hatching apparatus was kept in a dark incubator at 28 °C, optimal for *M. incognita* hatching. Freshly hatched J2 were collected daily from the water reservoir for 7 days. Only actively motile J2 harvested within 24 h of hatching were used for plant inoculation to ensure uniform infectivity and developmental synchrony.

#### Nematode Species Identification and Population Characterization

*M. incognita* race 1, a common race in China [25], was originally isolated from infected tomato (*Solanum lycopersicum* L.) plants. To establish a genetically uniform population for this study, a single egg mass was extracted from an infected tomato root and used to initiate a clonal line on the highly susceptible tomato cultivar ‘DaHong’ in a temperature-controlled greenhouse maintained at 25 °C ± 2 °C. This single egg-mass-derived population ensures genetic uniformity and eliminates virulence variation that can occur in mixed nematode populations. Species identity and race determination were confirmed in our laboratory using a combination of host-range testing and molecular markers, following the methodology described by Li et al. [26]. The clonal population was continuously maintained on ‘DaHong’ tomato through successive generations, with subculturing every 35–45 days to maintain population vigor and ensure consistent infectivity throughout the experimental period. For each subculture and experimental inoculation, egg inoculum was collected from nematode-infected roots using the sodium hypochlorite extraction method. Infected roots were gently uprooted from pots, thoroughly washed with tap water to remove adhering soil, cut into 2–3 cm pieces, and placed in a flask containing 0.5% sodium hypochlorite (NaOCl) solution. The flask was vigorously shaken for 5 min to digest the gelatinous matrix surrounding egg masses and release individual eggs [27]. The egg suspension was immediately filtered through nested sieves (200-mesh, 75 μm, and 500-mesh, 25 μm) to collect eggs, which were then rinsed thoroughly with sterile distilled water to remove residual NaOCl. Eggs were quantified under a stereomicroscope (Olympus SZX16, Tokyo, Japan) at 40× magnification using a counting chamber, and only preparations containing >95% viable, embryonated eggs were used in experiments.

### 2.3. Experimental Design and Plant Inoculation

All alfalfa seeds were surface-sterilized in a 0.5% NaOCl solution for 10 min with gentle agitation, then rinsed five times with sterile distilled water to remove residual disinfectant. Seeds were planted individually in 10 cm diameter plastic pots (one seed per pot) containing autoclaved sandy soil mixture (sand: soil ratio = 2:1 *v*/*v*). The sandy soil substrate was selected to facilitate nematode movement, root penetration during harvest, and subsequent egg extraction. Pots were arranged in a completely randomized design within a growth chamber (HP250GS-C, Ruihua, Wuhan, China) programmed to maintain 24 ± 2 °C with a 16 h light period (200 μmol m^−2^ s^−1^ photosynthetically active radiation provided by LED lamps) and 8 h of darkness. Plants were watered as needed to maintain consistent soil moisture without waterlogging. Five weeks after sowing, when seedlings had developed 4–6 true leaves and strong root systems, plants were inoculated with *M. incognita* second-stage juvenile (J2). The inoculum concentration was measured using a counting chamber under a stereomicroscope, and suspensions were adjusted to deliver exactly 500 J2 per plant in 5 mL sterile water. Inoculation was performed by pipetting the nematode suspension into two holes (2 cm deep, 2 cm from the stem base) created in the soil on opposite sides of each plant. The holes were immediately covered with soil to prevent J2 desiccation and ensure movement into the root zone. Four replicate plants were used for each cultivar, and the entire experiment was conducted twice (two independent experimental runs) to confirm reproducibility. The susceptible tomato cultivar DaHong was included as a positive control in each run to verify nematode infectivity and virulence.

### 2.4. Assessment of Resistance Indices

#### 2.4.1. Galling Index and Disease Index

At five weeks after inoculation (ten weeks after sowing), all plants were carefully removed from pots, and root systems were gently washed under running tap water to remove adhering soil while minimizing root damage. Root galling severity was assessed using a modified 0–5 scale adapted from Taylor and Sasser [28]: 0 = no visible galls or evidence of infection; 1 = 1–10% of root tissue showing galls; 2 = 11–20% galled tissue; 3 = 21–50% galled tissue; 4 = 51–80% galled tissue; and 5 = 81–100% of the root system galled. Each plant was given a single galling index (GI) score based on visual assessment of the entire root system by three independent evaluators, with the median score recorded.

Disease Index for each cultivar was calculated using the formula:

DI = (Σ(GI × Ni)/(N × 5)) × 100

where GI represents the galling severity level (0–5), Ni is the number of plants receiving that particular GI score, N is the total number of plants evaluated for that cultivar (n = 4), and 5 is the maximum possible GI score.

Based on DI values, varieties were classified into resistance categories: 0 = immune (I); 0–10 = highly resistant (HR); 11–20 = resistant (R); 21–40 = moderately resistant (MR); 41–60 = susceptible (S); and >60 = highly susceptible (HS).

#### 2.4.2. Egg Mass Staining and Counting

Following the galling assessment, roots were stained to visualize nematode egg masses. The number of egg masses was counted after root staining with erioglaucine, and egg mass density was calculated from root weight using the nondestructive technique of Omwega et al. [29]. Entire root systems were immersed in 0.015% (*w*/*v*) aqueous erioglaucine disodium salt solution (Acid Blue 9, FD&C Blue No. 1, C.I. 42090; Sigma-Aldrich, St. Louis, MO, USA, catalog no. E4505) for 20 min at room temperature with gentle agitation every 5 min to ensure uniform dye penetration. Erioglaucine selectively binds to the gelatinous matrix surrounding nematode egg masses, rendering them bright blue against unstained root tissue. After staining, roots were briefly rinsed in tap water to remove excess dye and examined under a stereomicroscope (Olympus SZX16, Tokyo, Japan). Root fresh weight (RFW) was recorded immediately after blotting excess water with paper towels. Egg masses (EM) on each root system were counted directly under the stereomicroscope (10–40× magnification) by systematically examining all root segments. Egg mass density was calculated as EM per gram RFW to normalize for differences in root size among varieties. The EMI was assigned to each plant based on total egg mass count, following the classification system of Taylor and Sasser (1978) [28]: 0 = no egg masses; 1 = 1–2 egg masses; 2 = 3–10 egg masses; 3 = 11–30 egg masses; 4 = 31–100 egg masses; and 5 = more than 100 egg masses. The average EMI for each cultivar was used to determine resistance categories: 0.0–1.0 = highly resistant (HR); 1.1–3.0 = resistant (R); 3.1–3.5 = moderately resistant (MR); 3.6–4.0 = slightly resistant (SR); and 4.1–5.0 = susceptible (S).

#### 2.4.3. Egg Number Quantification

For accurate measurement of nematode reproductive output, eggs were extracted from root systems using the same procedure described for inoculum preparation. After counting egg masses, roots were cut into small segments and treated with a 1% NaOCl solution in a reciprocating shaker for 3 min. The egg suspension was filtered through 200-mesh and 500-mesh sieves, and eggs collected on the 500-mesh sieve were transferred to a counting chamber. The total egg number (EN) per root system was calculated by counting three 1 mL aliquots of the thoroughly mixed egg suspension and multiplying by the total suspension volume.

### 2.5. Infection Rate and Larval Development Assessment

To study resistance mechanisms, a subset of varieties representing resistant (Gannong No. 9) and susceptible (Catera, Nongjing No. 1, WL168HQ) phenotypes were used in time-course infection experiments. Plants were inoculated with 500 J2 as described earlier, and root systems were collected at 2 days post-infection (dpi) to measure infection rates and at 7 dpi to evaluate larval development. For both timepoints, roots were carefully washed in sterile distilled water and stained with acid fuchsin to visualize nematodes inside the root tissues. Roots were cleared by autoclaving at 121 °C for 3 min in 1% NaOCl solution, then rinsed thoroughly with tap water. The cleared roots were placed in an acid fuchsin staining solution (0.035% acid fuchsin in a 1:1 glycerol: water mixture with 0.01% acetic acid) and heated in a water bath at 90 °C for 20 min. Excess stain was removed by destaining in acidified glycerol (glycerol:1% HCl, 1:1) at room temperature for 12–24 h until the background tissue was clear, but the nematodes remained dark red. Stained roots were mounted on glass slides, covered with coverslips, and examined systematically under a compound microscope (Olympus BX53, Tokyo, Japan) at 100–400× magnification. At two dpi, all J2 nematodes within the root tissues were counted to determine the infection rate (number of nematodes per root system). At 7 dpi, nematodes were counted and staged as J2, J3 (third-stage juvenile), or J4 (fourth-stage juvenile) based on morphological features like body size, stylet length, and gonad development. The percentage of nematodes in each stage was calculated for each plant.

### 2.6. Statistical Analysis

Statistical analyses were performed using R software (version 4.2.0; R Core Team , Vienna, Austria). The experiment followed a completely randomized design across two independent runs. Galling index data (ordinal scale 0–5) were analyzed using Kruskal–Wallis rank-sum tests with effect sizes quantified by epsilon-squared (ε^2^). Post hoc pairwise comparisons were performed using Dunn’s test with a Bonferroni correction (FSA package V0.10.0). Disease Index was calculated as: DI = (Σ(GI × Ni)/(N × 5)) × 100, where GI is galling severity (0–5), Ni is the number of plants with each score, N is the total plants, and 5 is the maximum score. Cultivars were classified as immune (DI = 0), highly resistant (0–10), resistant (11–20), moderately resistant (21–40), susceptible (41–60), or highly susceptible (>60). Egg mass and egg number data exhibited overdispersion (dispersion parameter >> 1) and were analyzed using generalized linear models with negative binomial distribution (glm.nb, MASS package V7.3-65). Models included cultivar as a fixed effect and experimental run as a covariate. Results are presented as estimated means with 95% confidence intervals, and pairwise comparisons are performed using estimated marginal means and Tukey adjustment (emmeans package V2.0.1). Cultivar × run interactions were tested using likelihood ratio tests. Non-significant interactions indicated consistent cultivar rankings across runs, justifying additive models. Linear mixed-effects models (lmer, lme4 package V1.1-38) included cultivar as a fixed effect and run as a random effect, with *p*-values from Satterthwaite approximation (lmerTest package V3.1-3). Intraclass correlation coefficients quantified the variance due to run effects. A composite resistance score (0–100 scale) was calculated by: (1) computing cultivar means for galling index, egg mass count, and egg number; (2) standardizing to z-scores; (3) inverting z-scores (multiplying by −1, since higher raw values indicate susceptibility); (4) averaging inverted z-scores; and (5) scaling to 0–100 (Score = 50 + (z × 10)), where 50 represents average resistance and each 10-point change represents one standard deviation. Resistance classification in this study is based primarily on Disease Index (DI), which is the standard method for assessing root-knot nematode resistance and allows direct comparison with published studies; Egg Mass Index (EMI) and composite scores are treated as complementary indicators that capture distinct biological aspects of the host-nematode interaction, with DI reflecting visible root damage and symptom severity while EMI specifically quantifies nematode reproductive success. Because these indices measure partially independent components of resistance (moderate correlation, r = 0.68), discrepant classifications can occur when cultivars exhibit different levels of symptom expression versus nematode reproduction; in such cases, DI provides the primary classification, while discrepancies may potentially reflect antibiosis (suppressed reproduction despite galling) versus tolerance (limited symptoms despite reproduction), though these remain putative mechanisms that would require direct experimental validation to confirm. Equal weighting was applied to galling index, egg mass count, and egg number in the composite score because each represents a distinct aspect of nematode impact: galling reflects feeding-site-induced anatomical damage, egg mass formation indicates successful female development and reproduction, and total egg number quantifies population growth potential. Throughout this manuscript, unless explicitly stated otherwise, the terms “resistant” and “susceptible” refer to DI-based classifications, while the composite score is used for overall cultivar ranking and comparative visualization. Correlations among resistance metrics were evaluated using Spearman’s rank correlation coefficient (rs) with cultivar-level means. Data are presented as median ± interquartile range (IQR) for ordinal data, estimated mean with 95% CI for count data, and mean ± SE for continuous data. All *p*-values were two-tailed with significance at α = 0.05. Multiple comparison adjustments-controlled family-wise error rates. Data visualization used ggplot2. Analysis code is available from the corresponding author upon request.

## 3. Results

### 3.1. Differential Resistance of Alfalfa Varieties Based on Disease Index

**Figure 2:**
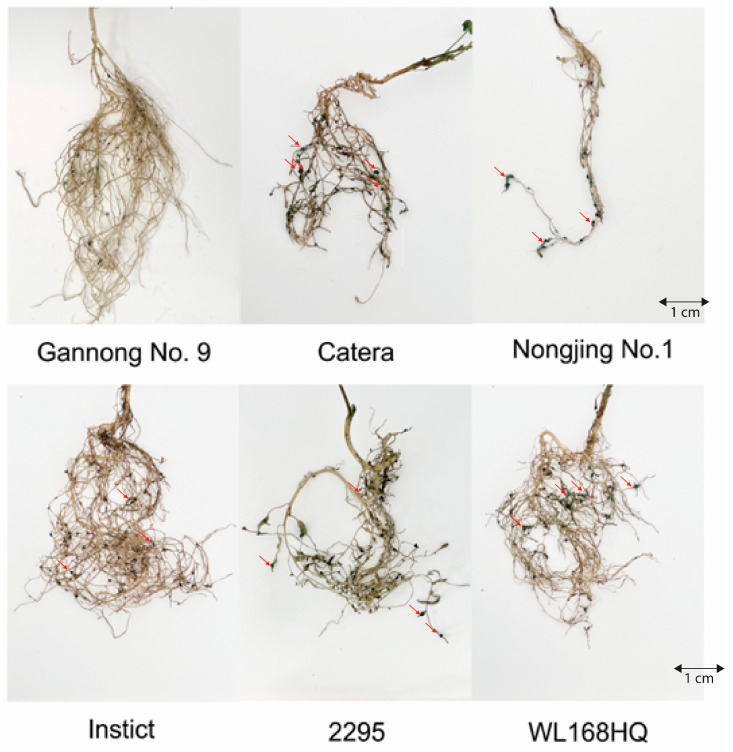
Root galling and egg mass production in susceptible alfalfa varieties infected with *M. incognita*. Representative root systems of susceptible alfalfa cultivars stained with erioglaucine (Acid Blue 9) to visualize nematode egg masses (appearing as bright blue structures, indicated by arrows). Scale bars = 1 cm.

Significant differences in nematode resistance were observed among the 24 alfalfa varieties tested. Based on the DI, which integrates galling severity across all plants and varieties, the varieties exhibited a wide range of resistance responses, from highly resistant to susceptible (Table 1). The most resistant variety was Gannong No. 9 (China), displaying highly resistant (HR) characteristics with a DI of 10.0%. Among the 24 varieties evaluated, 16 (66.7%) were classified as resistant (R) or highly resistant (HR) based on DI ≤ 20%, indicating that a substantial proportion of the tested germplasm possesses valuable resistance traits. These included varieties from diverse geographical origins: China (Gannong No. 9, Jiuquan), America (Moste, Dryland, Kangsai 1, among others), Canada (3010, 5020), and Australia (Aurora). Six varieties were classified as moderately resistant (MR, DI = 20–40%), with DI values ranging from 22.5% to 40.0%. Only two varieties were classified as susceptible (S): Catera and Nongjing No. 1. Notably, Nongjing No. 1 showed the highest susceptibility among all varieties tested, with extensive root damage characterized by severely reduced lateral root development (Figure 2). Erioglaucine staining clearly revealed egg masses adhered to the root surfaces of susceptible varieties (Figure 2). Catera, 2295, Instict, and WL168HQ displayed extensive root nodulation with numerous stained egg masses visible on their root systems, confirming active nematode reproduction in these genotypes.

### 3.2. Nematode Reproduction Parameters and Root Biomass Responses

Nematode reproduction varied dramatically among alfalfa varieties, with total egg production ranging from complete suppression to severe infestation (Table 2). Gannong No. 9 exhibited exceptional resistance, with zero egg production, while the most susceptible variety, Nongjing No. 1, produced 7904 eggs per plant, representing more than a 3-log difference in reproductive success. The top-performing varieties achieved 98–100% reductions in egg production compared with the susceptible control: Dryland (200 eggs, 99.2% reduction), Kangsai 1 (212 eggs, 99.1% reduction), and Moste (460 eggs, 98.1% reduction).

Egg mass density per gram root fresh weight ranged from 3.95 masses/g (Dryland) to 68.06 masses/g (Nongjing No. 1). Based on the EMI, only Gannong No. 9 achieved highly resistant (HR) status, while 22 varieties were classified as resistant, and one variety (2295) as moderately resistant. Notably, several varieties showed discrepant classifications between DI and EMI. For example, Catera and Nongjing No. 1 were classified as susceptible by both indices, whereas varieties such as Jiuquan showed minimal galling but supported substantial egg production. Root fresh weight (RFW) varied from 0.33 g (Aohan) to 0.92 g (WL343HQ) at 5 weeks post-inoculation, but showed no significant correlation with galling severity, suggesting root biomass alone does not determine resistance or susceptibility. Some highly infected varieties maintained vigorous growth: WL343HQ produced the highest root biomass (0.92 g) despite supporting 2827 eggs, while Instict maintained 0.80 g despite 6658 eggs, indicating tolerance mechanisms that compensate for nematode damage through continued growth.

### 3.3. Comparison of Resistance Assessment Methods: DI Versus EMI

**Figure 3:**
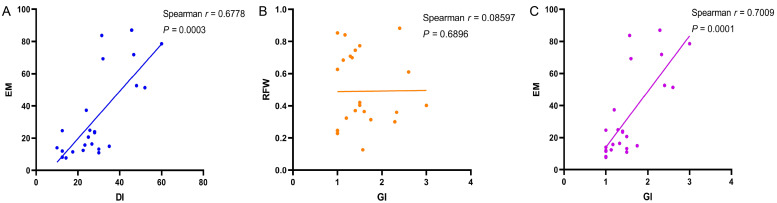
Correlation analysis among resistance metrics for 24 alfalfa cultivars infected with *M. incognita* at 5 weeks post-inoculation. (**A**) Disease Index (DI, %) vs. Egg Mass Index (EMI, 0–5 scale) showing moderate positive correlation (Spearman’s rs = 0.68, *p* < 0.001). The moderate rather than strong correlation indicates that galling severity and egg mass production represent partially independent components of the resistance phenotype. (**B**) Galling Index (GI, 0–5 ordinal scale) vs. root fresh weight (RFW, g) showing no significant correlation (rs = 0.09, *p* = 0.68), indicating that root biomass accumulation occurs independently of nematode infection severity and is not a reliable resistance indicator. (**C**) Egg mass count per gram RFW vs. Galling Index showing strong positive correlation (rs = 0.70, *p* < 0.001), confirming that visible root damage generally corresponds with nematode reproductive success. Each point represents one cultivar mean (n = 4 replicates per cultivar). Shaded areas indicate 95% confidence intervals for fitted regression lines.

Significant discrepancies appeared when comparing resistance classifications from DI versus EMI assessments. Both indices aim to measure nematode-plant interactions, but they agreed on only 15 of 24 varieties (62.5%), with 9 varieties (37.5%) showing conflicting resistance ratings (Table 3). The most notable differences occurred with varieties identified as susceptible by DI but classified as resistant or moderately resistant by EMI. For instance, varieties 2295, Instict, and WL168HQ exhibited high root galling (DI = 47–52, labeled as Susceptible) but had moderate egg mass burdens (EMI = 3.40–4.00), leading to reclassification as Slightly or Moderately Resistant under EMI criteria. Conversely, Gannong No. 9, initially rated as Highly Resistant based on DI (DI values of 0–10), was downgraded to Resistant under EMI classification. Among resistance cultivars, Jiuquan showed the largest discrepancy, receiving a Resistant rating by DI (12.5) but only a Moderately Resistant rating by EMI (3.40), indicating that despite minimal galling, this variety still permitted moderate egg mass production. Correlation analysis revealed that DI and EMI were significantly positively related (Spearman’s r = 0.68, *p* < 0.001; Figure 3A), indicating these indices measure related aspects of the plant-nematode interaction. However, the moderate rather than strong correlation (r^2^ = 0.46, indicating 46% shared variance) accounts for the classification inconsistencies observed across several varieties. This suggests that root galling and nematode reproductive output, while correlated, represent partially independent components of the resistance phenotype. Root fresh weight showed no significant correlation with galling index (Spearman’s r = 0.09, *p* = 0.68; Figure 3B), indicating that root biomass accumulation occurs independently of nematode infection severity. Some resistant varieties, such as Jiuquan and 310SC, maintained robust root systems (mean root weight >0.7 g) despite nematode challenge, while certain susceptible varieties, like Instict, also exhibited substantial root biomass (0.81 g). This independence of root weight from resistance status demonstrates that root biomass is not a reliable indicator of resistance and should not be used as a primary screening criterion. In contrast, egg mass count per gram root weight showed a strong positive correlation with galling index (Spearman’s r = 0.70, *p* < 0.001; Figure 3C), confirming that visible root galling generally corresponds with successful nematode reproduction.

### 3.4. Preliminary Observations on Nematode Infection and Early Development

**Figure 4:**
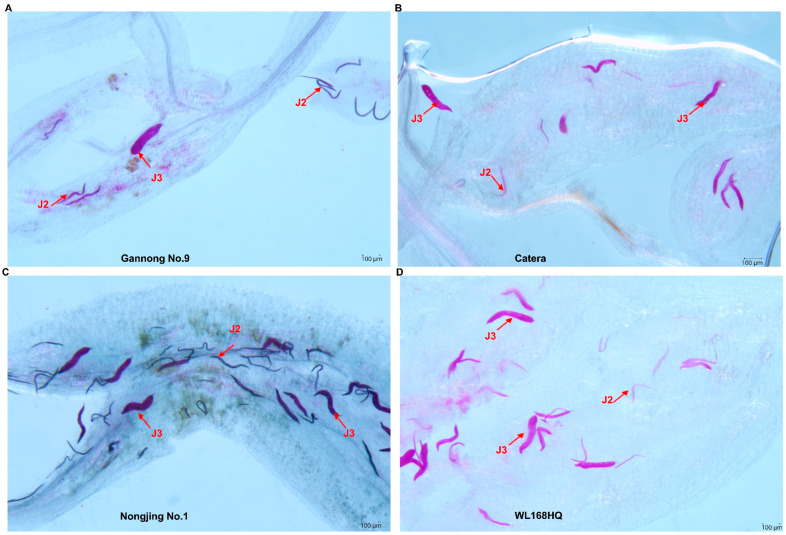
Developmental stages of *M. incognita* within alfalfa roots at 7 days post-inoculation. Developmental stages of *M. incognita* visualized by acid fuchsin staining at 7 days post-inoculation (dpi). (**A**) Gannong No. 9 (resistant variety) showing predominantly second-stage juveniles (J2) with minimal progression to third-stage juveniles (J3). (**B**–**D**) Susceptible varieties (Catera, Nongjing No. 1, WL168HQ) showing mixed populations of J2 and J3. Nematodes appear as dark red structures within cleared root tissues (indicated by red arrows). Scale bars = 100 μm.

**Figure 5:**
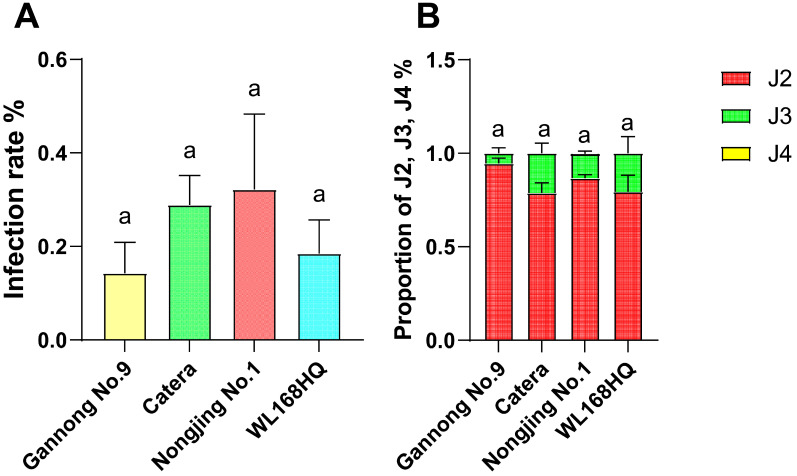
Nematode infection dynamics and developmental stage distribution in resistant and susceptible alfalfa cultivars. (**A**) Infection rate (%) at 2 days post-inoculation (dpi). Bars represent mean ± SE (n = 4 plants per cultivar). No statistically significant differences were observed among cultivars (Kruskal–Wallis test, *p* > 0.05), indicating that nematode penetration occurred at comparable frequencies across resistance classifications at this early timepoint. (**B**) Proportion of juvenile nematodes at developmental stages J2 (second-stage juveniles, red), J3 (third-stage juveniles, green), and J4 (fourth-stage juveniles, yellow) at 7 dpi. Bars represent mean proportions (n = 4 biological replicates per cultivar). Statistical analysis revealed no significant differences in J2 proportions among cultivars (Kruskal–Wallis test followed by post hoc pairwise comparisons with Bonferroni correction, *p* > 0.05). Shared letters above bars indicate statistical groupings; all cultivars share the same letter (a), indicating no significant differences. The predominance of J2 larvae across all genotypes (78–89%) and high within-cultivar variability suggest that 7 dpi represents an early infection phase where resistance-associated effects on development, if present, are not yet strongly expressed.

To explore potential differences in infection dynamics between resistant and susceptible alfalfa cultivars, nematode penetration and early larval development were examined in one resistant cultivar (Gannong No. 9) and three susceptible cultivars (Catera, Nongjing No. 1, and WL168HQ) (Figure 4A–D). At 2 days post-inoculation (dpi), no statistically significant differences in infection rates were detected among the four cultivars (Figure 5A). Infection percentages ranged from 15.0 ± 4.8% in Gannong No. 9 to 32.0 ± 15.7% in Nongjing No. 1, with intermediate values observed in Catera (28.0 ± 8.1%) and WL168HQ (19.0 ± 7.9%). The substantial overlap among cultivars and the relatively high variability indicate that initial nematode penetration occurred at comparable frequencies across resistance classifications. Larval developmental progression was assessed at 7 dpi through acid fuchsin staining and stage classification (Figure 5B). Across all cultivars, the majority of nematodes remained as second-stage juveniles (J2): Gannong No. 9 (89.4 ± 8.4% J2, 10.6 ± 8.4% J3), Catera (78.8 ± 11.9% J2, 21.2 ± 11.9% J3), Nongjing No. 1 (88.3 ± 3.6% J2, 11.7 ± 3.6% J3), and WL168HQ (78.4 ± 19.7% J2, 21.6 ± 19.7% J3). Fourth-stage juveniles (J4) were essentially absent across all cultivars (0–1.5%). Statistical analysis revealed no significant differences in J2 proportions among cultivars (*p* > 0.05). The high within-variety variability (e.g., WL168HQ SD = 19.7%) and overlapping distributions between resistant and susceptible groups indicate that 7 dpi is too early to detect clear developmental differences. These findings suggest that: (1) our single early timepoint is insufficient to characterize resistance mechanisms, (2) developmental arrest, if present, may only become apparent at later stages (14–35 dpi), or (3) resistance operates through mechanisms other than developmental inhibition. Further investigation with multiple timepoints, larger sample sizes, and histological examination of feeding site development is required to elucidate resistance mechanisms in alfalfa.

### 3.5. Comprehensive Resistance Profiling Across Geographic Origins

**Figure 6:**
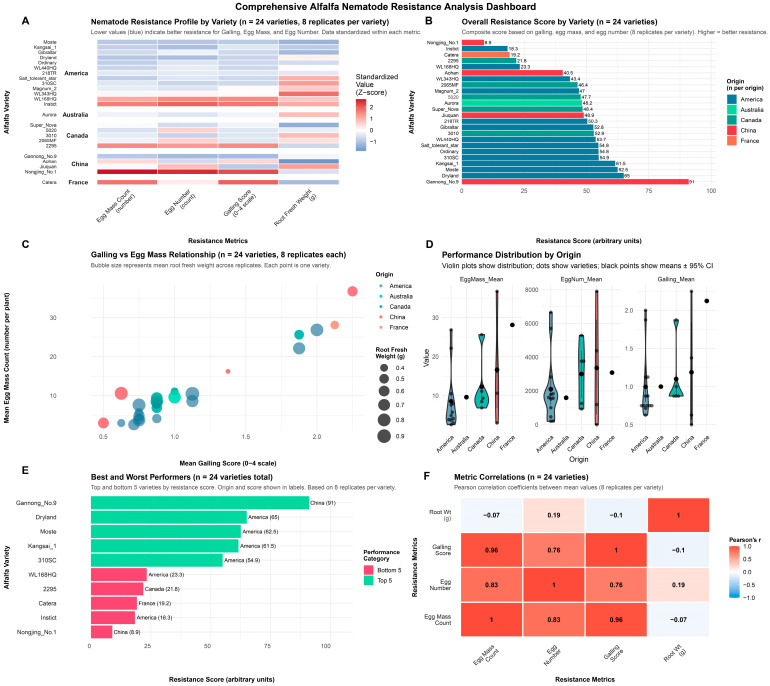
Comprehensive resistance analysis of 24 alfalfa cultivars against *M. incognita*. (**A**) Heatmap of standardized resistance metrics (z-scores) showing blue = resistant (negative z-scores) and red = susceptible (positive z-scores). Varieties ordered by composite resistance score (left). (**B**) Composite resistance scores (0–100 scale) ranked by performance, with higher values indicating greater resistance. The dashed line at 50 represents the population mean. (**C**) Scatter plot of mean Galling Index vs. egg mass count per gram RFW; bubble size proportional to root fresh weight (g). Each point represents the mean of one variety (n = 4 per variety). (**D**) Violin plots showing the distribution of resistance metrics by country of origin. Box plots inside violins show median (center line), IQR (box), and range (whiskers). Sample sizes: USA n = 14, Canada n = 5, China n = 4, Australia n = 1, France n = 1. (**E**) Top 5 resistant varieties (left, blue) and bottom 5 susceptible varieties (right, red) based on composite scores. (**F**) Correlation matrix of all measured variables (n = 24). Circle size and color intensity indicate the strength of the correlation. red = positive, blue = negative. All correlations shown are Spearman’s rank coefficients (rs).

Combining multiple resistance metrics into a composite score uncovered significant differences in overall resistance performance (Figure 6A,B). Scores ranged from 8.9 to 91 on a 0–100 scale. The highest-performing varieties by composite score were Gannong No. 9 (91), Dryland (65), and Moste (62.5). In terms of formal DI classification, Gannong No. 9 was highly resistant (HR, DI: 10%), while Dryland and Moste were resistant (R, DI: 14.3% and 12.5%, respectively). These leading varieties consistently showed low values across all pathogen-related metrics in the standardized heatmap (Figure 6A), with blue indicating strong resistance. In contrast, the five lowest-scoring varieties by composite score were Nongjing No. 1 (China, 8.9), Instinct (America, 18.3), Catera (France, 19.2), 2295 (Canada, 21.8), and WL168HQ (America, 23.3). By DI classification, Nongjing No. 1 and Catera were susceptible (S), while 2295, Instinct, and WL168HQ were moderately resistant (MR), illustrating discrepancies between galling severity and overall pathogen burden. The scatter plot analysis examining the relationship between mean galling score and mean egg mass production revealed a positive trend, though with considerable variation (Figure 6C). Most varieties clustered in the low-galling (0.5–1.5) and low-egg-mass (10–40 masses/g) regions, reflecting that resistant genotypes are predominant in the tested panel. Several susceptible varieties appeared in the high-severity zone: Nongjing No. 1 exhibited the highest egg mass count (>80 per gram RFW) with high galling scores (>2.5), while Catera, 2295, and Aohan also displayed elevated egg production. Root fresh weight showed no clear association with resistance status, with both resistant and susceptible varieties displaying a wide range of root biomass values.

Analysis of resistance patterns by country of origin revealed distinct geographic trends (Figure 6D). American varieties (n = 13) exhibited the broadest performance range, including both highly resistant varieties such as Dryland and Moste and susceptible ones such as Instinct and WL168HQ, indicating significant genetic diversity within American germplasm. Canadian varieties (n = 5) demonstrated moderate variation with slightly higher pathogen loads than the top performers. Chinese varieties (n = 4) exhibited a clear bimodal distribution, clearly separating resistant varieties (Gannong No. 9, Kangsai 1, Jiuquan, Aohan) from susceptible varieties (Nongjing No. 1). The single tested French variety, Catera, consistently performed poorly across all resistance metrics.

A comprehensive correlation matrix analysis was conducted to quantify relationships among all measured resistance parameters (Figure 6F). Strong positive correlations were noted among pathogen-related metrics, with egg mass count and egg number exhibiting the highest correlation (r = 0.96), indicating these metrics nearly reflect the same aspect of nematode reproductive success. Galling scores showed strong positive correlations with both egg mass (r = 0.96) and egg number (r = 0.83), confirming that visible root damage reliably indicates nematode establishment and reproduction. Conversely, root fresh weight showed minimal correlations with pathogen metrics: weak negative correlations with egg mass (r = −0.10) and egg number (r = −0.07), and a weak positive correlation with galling (r = 0.19). These weak correlations support the observation that root biomass accumulation occurs independently of nematode infection severity, suggesting RFW should not serve as the primary resistance indicator.

## 4. Discussion

### 4.1. Variation in Resistance Among Alfalfa Cultivars

Substantial variation in *M. incognita* resistance was observed among the 24 alfalfa cultivars evaluated. Nineteen exhibited resistances, and five were susceptible based on the Disease Index. This genetic diversity provides valuable resources for breeding nematode-resistant cultivars. Gannong No. 9 ranked highest across multiple criteria (DI = 10.0%, composite score = 91, zero egg production). It serves as valuable germplasm for direct deployment in infested regions and as a parent in breeding programs. Other promising cultivars include U.S. varieties Dryland, Moste, and Kangsai 1, which were classified as resistant (R) by DI and showed strong performance across multiple indices. In contrast, widely cultivated varieties Nongjing No. 1 (China) and Catera (France) were classified as susceptible (S) by DI. They exhibited extensive root galling and substantial nematode reproduction (7904 and 3088 eggs per plant, respectively). Identifying susceptible cultivars within commercial germplasm underscores the need for systematic resistance screening before variety deployment in nematode-endemic regions. Resistance is not universal across alfalfa germplasm, making careful variety selection essential for sustainable production.

Alfalfa differs fundamentally from the annual diploid model systems, where most nematode resistance mechanisms have been characterized [30]. As a perennial autotetraploid species with a nitrogen-fixing symbiosis, its resistance pathways may differ substantially from those of crops like tomato (*Mi-1* gene) or potato (*Hero A* gene) [31]. The autotetraploid genome may produce quantitative rather than qualitative resistance phenotypes. Additionally, symbiotic relationships with *Sinorhizobium meliloti* could modulate defense responses differently than in non-symbiotic crops [32]. Direct investigation of alfalfa-specific mechanisms through histology, genetic mapping, and molecular characterization is essential, rather than assuming that mechanisms are conserved from distant crop relatives.

### 4.2. Assessment of Resistance Using Multiple Complementary Indices

DI and EMI showed moderate correlation (r = 0.68, *p* < 0.001), indicating these indices measure related but partially independent resistance components. Resistance classification in this study is based primarily on DI, the standard method in plant nematology that enables direct comparison with published evaluations [8]. EMI and total egg counts provide complementary information on reproductive suppression, which is essential for a comprehensive assessment [33,34].

Discrepancies between DI and EMI classifications occurred in 9 of 24 cultivars (37.5%), reflecting biologically meaningful differences. Cultivars classified as moderately resistant to susceptible by DI but resistant by EMI (2295, Instict, WL168HQ) exhibit substantial galling but suppress egg mass production relative to their galling severity. This pattern is consistent with what would be expected from antibiosis—a mechanism that permits feeding-site formation but interferes with subsequent female maturation or fecundity. However, this interpretation remains hypothetical. Direct confirmation through histological examination of feeding sites and giant cell morphology, assessment of female developmental success and fecundity rates, measurements of nematode viability within roots, and molecular analyses of defense gene expression would be required to substantiate whether antibiosis is the operative mechanism in these cultivars. Conversely, cultivars showing minimal galling but moderate reproduction may reflect incomplete resistance or differences in how symptom expression and reproductive success are coupled in different genetic backgrounds. These patterns suggest hypotheses for further investigation rather than established mechanisms.

The DI assesses field performance, stand persistence, and root health, which are critical for perennial alfalfa, where root integrity impacts longevity and yield. EMI captures nematode population dynamics and potential impact on subsequent crops in rotation systems. For breeding programs, we recommend an integrated approach that includes primary screening using DI for standardized, rapid assessment, followed by secondary confirmation using egg mass counts or total egg enumeration to validate reproductive suppression. Composite scoring that integrates multiple parameters enables overall cultivar ranking, while mechanistic studies on selected resistant and susceptible cultivars can elucidate resistance pathways. This multi-index framework captures the full spectrum of host responses and enables identification of germplasm with durable, multifaceted resistance.

Root fresh weight showed no correlation with galling (r = 0.09, *p* = 0.68) or nematode reproduction (r = −0.10 with egg mass, r = −0.07 with egg number). Both resistant and susceptible cultivars maintained substantial biomass, likely reflecting genetic variation in inherent growth vigor, the early assessment timing (5 weeks post-inoculation) before cumulative damage becomes pronounced, and potential tolerance mechanisms. Root biomass is not a reliable indicator of resistance for screening programs.

### 4.3. Preliminary Observations on Early Infection Dynamics

To explore potential differences in infection dynamics between resistant and susceptible alfalfa cultivars, nematode penetration and early larval development were examined in one resistant cultivar (Gannong No. 9) and three susceptible cultivars (Catera, Nongjing No. 1, and WL168HQ) at 2 and 7 days post-inoculation (dpi).

No statistically significant differences in infection rates were detected among the four cultivars at 2 dpi (Figure 5A). Infection percentages ranged from 15.0 ± 4.8% in Gannong No. 9 to 32.0 ± 15.7% in Nongjing No. 1, with intermediate values in Catera (28.0 ± 8.1%) and WL168HQ (19.0 ± 7.9%). These data indicate that under our experimental conditions, nematode penetration occurred at comparable frequencies across resistance classifications. This pattern is compatible with post-penetration resistance mechanisms documented in other plant-nematode systems [2,35,36,37], but our limited sampling design (n = 4 per cultivar, single timepoint, no normalization to root surface area) means these data cannot definitively establish whether resistance operates before or after penetration. Comprehensive time-course analyses with larger sample sizes and normalized measurements would be required to resolve the timing of resistance expression.

Larval developmental progression was assessed at 7 dpi through acid fuchsin staining and morphological stage classification (Figure 4 and Figure 5B). Across all cultivars, the majority of nematodes remained as second-stage juveniles (J2): Gannong No. 9 (89.4 ± 8.4% J2, 10.6 ± 8.4% J3), Catera (78.8 ± 11.9% J2, 21.2 ± 11.9% J3), Nongjing No. 1 (88.3 ± 3.6% J2, 11.7 ± 3.6% J3), and WL168HQ (78.4 ± 19.7% J2, 21.6 ± 19.7% J3). Fourth-stage juveniles (J4) were essentially absent across all cultivars (0–1.5%). Statistical analysis revealed no significant differences in J2 proportions among cultivars (Kruskal–Wallis test, *p* > 0.05). Although susceptible cultivars exhibited numerically higher proportions of third-stage juveniles (J3: 21–22%) compared to Gannong No. 9 (10.6% J3), the high within-cultivar variability and overlapping distributions prevented detection of statistically significant differences.

These preliminary observations from a single early timepoint with modest sample sizes (n = 4 biological replicates per cultivar) are insufficient to characterize resistance mechanisms or determine the timing of resistance expression. The absence of statistically significant developmental differences at 7 dpi does not allow conclusions about whether resistant cultivars suppress nematode development, as this pattern could reflect several non-exclusive scenarios. First, resistance mechanisms may operate primarily during later developmental stages (14–35 dpi) when feeding-site establishment, giant-cell maturation, and female reproduction occur, meaning seven days post-infection may be too early to detect resistance-associated developmental inhibition. Second, resistance may involve effects on nematode fitness, viability, or fecundity that do not manifest as overt developmental arrest visible through stage classification, with resistant cultivars potentially permitting normal early development while compromising subsequent female maturation or egg production. Third, the modest sample sizes (n = 4) and high biological variability may have prevented detection of genuine developmental differences that exist but are subtle at this early timepoint. Fourth, resistance in Gannong No. 9 could involve tolerance mechanisms or post-reproductive population suppression rather than developmental inhibition, in which case no early developmental differences would be expected. Distinguishing among these possibilities requires comprehensive time-course studies with sampling at multiple timepoints (7, 14, 21, 35 dpi), histological examination of feeding-site morphology and giant-cell integrity, direct assessment of nematode viability and sex ratios within roots, quantification of female developmental success and fecundity rates, and molecular profiling of defense gene expression at multiple infection stages. Until such evidence becomes available, the mechanistic basis of resistance in alfalfa remains unresolved.

While our early infection dynamics observations cannot resolve mechanisms, they should not be dismissed as uninformative. The consistent and substantial suppression of nematode reproduction observed in resistant cultivars such as Gannong No. 9 across two independent experimental runs (zero egg production versus 7904 eggs in susceptible controls) provides robust evidence that exploitable genetic variation for resistance exists within alfalfa germplasm. This reproducible phenotypic variation, regardless of its underlying mechanism, represents valuable genetic material for breeding programs. The present study should be interpreted as providing a phenotypic framework for resistance assessment and identifying germplasm resources, rather than offering a mechanistic resolution of resistance pathways. Future mechanistic investigations building on this phenotypic foundation will be essential to understanding how resistant cultivars achieve the observed suppression of nematode establishment and reproduction.

### 4.4. Study Limitations and Future Research Directions

Several constraints should be considered when interpreting these results. First, evaluations used four replicates per cultivar, which provides preliminary screening data but may not fully capture within-cultivar genetic variation in heterogeneous autotetraploid populations. Second, assessments employed a single *M. incognita* race 1 population under controlled conditions (24 ± 2 °C). Resistance expression can vary with environmental factors (temperature, soil type, moisture) and nematode virulence. Resistance rankings should be considered environment- and population-specific until validated under field conditions against geographically diverse populations. Third, developmental analysis examined a limited set of cultivars at early timepoints (2 and 7 dpi) with modest sample sizes, yielding no significant differences. As discussed in Section 4.3, these preliminary observations were insufficient to characterize resistance mechanisms. Fourth, the 5-week assessment captured only initial responses and a single nematode generation, not long-term resistance stability.

Future research priorities include multi-location field trials across diverse environments and nematode populations, including other Meloidogyne species, assessment of resistance durability over multiple growing seasons, and evaluation under abiotic stress conditions that could compromise resistance. Larger-scale screening with increased replication is needed, along with comprehensive mechanistic investigations including time-course analyses of nematode development, histological examination of feeding sites, transcriptomic profiling, and genetic mapping to identify resistance loci. Long-term field studies assessing practical benefits for yield stability and nematode population management will be essential to translate these findings into practical breeding outcomes.

Despite these limitations, this study provides resistance rankings across commercially important alfalfa germplasm, identifies elite resistant cultivars suitable for breeding and deployment, establishes an integrated multi-index framework for future screening programs, and highlights the distinction between symptom-based resistance and reproductive suppression that should inform breeding strategies for developing durable, comprehensive resistance to root-knot nematodes in alfalfa.

## 5. Conclusions

This comprehensive screening of 24 alfalfa varieties for resistance to *M. incognita* reveals considerable genetic variation within current commercial germplasm, with 19 of 24 varieties exhibiting slight to high resistance, while 5 were susceptible. The discovery of highly resistant varieties, such as Gannong No. 9, provides valuable germplasm resources for immediate application in infested regions and for integration into long-term breeding programs. Our preliminary mechanistic study suggests that resistance may primarily function through post-penetration developmental inhibition rather than by preventing nematode entry, though additional histological analyses and feeding-site observations are needed to confirm this mechanism. These initial findings offer insights for future molecular research. Notably, analysis of the DI and EMI shows a moderate correlation but significant differences in classification across several varieties, emphasizing that a comprehensive resistance assessment should employ multiple metrics rather than relying on a single index. This study provides a comprehensive phenotypic framework for resistance assessment and identifies valuable germplasm resources, rather than offering a mechanistic resolution of resistance pathways. The consistent suppression of nematode reproduction observed in resistant cultivars across two independent experimental runs demonstrates exploitable genetic variation suitable for breeding programs. However, the mechanistic basis of this resistance, including the timing of resistance expression, cellular responses at feeding sites, and molecular pathways involved, remains to be elucidated through targeted histological, developmental, and molecular investigations. Future studies combining time-course infection analyses, microscopic examination of giant cell formation, transcriptomic profiling, and genetic mapping will be essential to understand how resistant cultivars suppress nematode establishment and reproduction.

## Figures and Tables

**Table 1:** Resistance of alfalfa cultivars to *Meloidogyne incognita* based on galling severity and Disease Index.

Rank	Variety	Origin	Galling (Median, IQR)	Disease Index (%)	Resistance Classification
1	Gannong No. 9	China	0.5 (0.0–1.0)	10.0%	HR
2	Moste	America	1.0 (0.0–1.0)	12.5%	R
3	Dryland	America	1.0 (0.5–1.0)	14.3%	R
4	Kangsai 1	America	1.0 (1.0–1.0)	17.5%	R
5	Ordinary	America	0.5 (0.0–1.0)	15.0%	R
6	Salt tolerant star	America	1.0 (0.8–1.0)	15.0%	R
7	310SC	America	0.5 (0.0–1.2)	15.0%	R
8	WL440HQ	America	0.5 (0.0–1.0)	15.0%	R
9	Gibraltar	America	1.0 (0.0–1.0)	15.0%	R
10	3010	Canada	1.0 (0.8–1.0)	17.5%	R
11	218TR	America	1.0 (0.0–1.2)	17.5%	R
12	Aurora	Australia	1.0 (0.8–1.2)	20.0%	R
13	Super Nova	Canada	1.0 (0.0–1.2)	20.0%	R
14	Magnum 2	America	1.0 (1.0–1.0)	22.5%	MR
15	5020	Canada	0.5 (0.0–1.2)	17.5%	R
16	Jiuquan	China	1.0 (0.0–1.0)	12.5%	R
17	2065MF	Canada	1.0 (0.0–1.2)	17.5%	R
18	WL343HQ	America	1.0 (1.0–1.2)	22.5%	MR
19	Aohan	China	1.0 (1.0–1.5)	27.5%	MR
20	Catera	France	2.0 (1.0–3.0)	42.5%	S
21	WL168HQ	America	1.5 (1.0–3.0)	37.5%	MR
22	2295	Canada	2.0 (1.0–3.0)	37.5%	MR
23	Instict	America	2.0 (1.0–2.5)	40.0%	MR
24	Nongjing_No. 1	China	2.0 (1.0–3.2)	45.0%	S

Resistance classification: HR (highly resistant, DI 0–10), R (resistant, 11–20), MR (moderately resistant, 21–40), S (susceptible, 41–60). Six varieties showed HR; five showed S.

**Table 2:** Nematode Reproduction Parameters and Root Characteristics of 24 Alfalfa Varieties at 5 Weeks Post-Inoculation with *M. incognita*.

Rank	Variety	RFW (g)	EM (per g RFW)	EMI Score	EN	EMI Class
1	Gannong No. 9	0.60 ± 0.50	5.00 ± 7.18	1.00	0.00 ± 0.00	HR
2	Moste	0.41 ± 0.23	7.32 ± 8.56	1.12	460.00 ± 536.66	R
3	Dryland	0.65 ± 0.58	3.95 ± 3.54	1.14	200.00 ± 447.21	R
4	Kangsai 1	0.48 ± 0.23	8.60 ± 7.42	1.50	212.00 ± 241.13	R
5	Ordinary	0.53 ± 0.19	7.32 ± 11.47	1.12	1575.00 ± 2868.98	R
6	Salt_tolerant_star	0.79 ± 0.25	4.75 ± 6.30	1.25	1613.33 ± 3145.51	R
7	310SC	0.83 ± 0.29	9.19 ± 12.29	1.50	1004.17 ± 1069.41	R
8	WL440HQ	0.63 ± 0.33	7.75 ± 16.98	1.12	1833.33 ± 1183.69	R
9	Gibraltar	0.43 ± 0.25	13.37 ± 25.44	1.25	2047.62 ± 3310.13	R
10	3010	0.77 ± 0.27	11.04 ± 9.31	2.00	929.17 ± 1479.00	R
11	218TR	0.56 ± 0.32	14.29 ± 18.30	1.75	1788.33 ± 2860.96	R
12	Aurora	0.77 ± 0.25	12.51 ± 11.27	2.00	1595.83 ± 1982.66	R
13	Super_Nova	0.40 ± 0.35	27.83 ± 30.00	2.00	1262.86 ± 2274.21	R
14	Magnum 2	0.74 ± 0.17	11.66 ± 9.16	2.12	1525.00 ± 1383.32	R
15	5020	0.58 ± 0.37	11.86 ± 17.74	1.12	3766.67 ± 2845.92	R
16	Jiuquan	0.83 ± 0.23	12.80 ± 10.96	1.88	4371.43 ± 3555.66	R
17	2065MF	0.69 ± 0.22	13.59 ± 15.89	1.62	3773.33 ± 5971.34	R
18	WL343HQ	0.92 ± 0.36	11.41 ± 14.67	2.00	2826.67 ± 4632.04	R
19	Aohan	0.33 ± 0.28	49.24 ± 50.30	2.50	1192.50 ± 1554.34	R
20	Catera	0.44 ± 0.23	63.93 ± 62.77	2.88	3088.00 ± 4316.80	R
21	WL168HQ	0.74 ± 0.23	29.91 ± 32.70	2.50	5695.83 ± 6226.02	R
22	2295	0.51 ± 0.24	50.25 ± 45.69	3.12	5286.66 ± 6813.82	MR
23	Instict	0.80 ± 0.17	33.58 ± 33.13	2.86	6657.57 ± 6333.09	R
24	Nongjing_No. 1	0.54 ± 0.21	68.06 ± 65.94	3.00	7904.17 ± 9424.86	R
-	Dahong (Control) ^†^	3.33 ± 0.21	32.23 ± 1.98	4.67	24,200.00 ± 1205.00	S

RFW = root fresh weight (g); EM = egg mass per gram root fresh weight; EMI Score = Egg Mass Index score (0–5 scale); EN = total egg number per plant; EMI Class = resistance classification based on EMI (HR = highly resistant, R = resistant, MR = moderately resistant, S = susceptible). ^†^ Dahong is a susceptible control variety included for comparison. Values represent mean ± standard deviation (n = 4 replicates). Note: Some varieties classified as MR or S by Disease Index (DI) show R classification by EMI, indicating suppression of nematode reproduction despite moderate galling severity.

**Table 3:** Comparison of resistance classifications based on Disease Index (DI) and Egg Mass Index (EMI).

Rank	Variety	Origin	Galling Index	Disease Index	DI Classification	Egg Mass Index	EMI Classification	Composite Score
1	Gannong No. 9	China	0.5 (0–1)	10.0	HR	1.80	R	91.0
2	Dryland	America	1.0 (0–1)	14.3	R	2.00	R	65.0
3	Moste	America	1.0 (0–1)	12.5	R	2.60	R	62.5
4	Kangsai 1	America	1.0 (1–1)	17.5	R	2.43	R	61.5
5	Salt-tolerant Star	America	1.0 (0–1)	12.5	R	1.60	R	60.2
6	Magnum 2	America	1.0 (1–1)	22.5	MR	2.50	R	56.8
7	Gibraltar	America	1.0 (0–1)	24.0	MR	2.80	R	55.3
8	310SC	America	1.0 (0–1)	25.0	MR	3.00	R	54.1
9	3010	Canada	1.0 (0–1)	23.3	MR	2.67	R	53.7
10	WL343HQ	America	1.0 (1–2)	25.7	MR	2.43	R	51.2
11	218TR	America	1.0 (0–2)	28.0	MR	3.00	R	49.8
12	2065MF	Canada	1.0 (0–2)	28.0	MR	3.20	MR	48.5
13	Aurora	Australia	1.0 (1–2)	26.7	MR	2.83	R	47.9
14	Ordinary	America	1.0 (0–1)	30.0	MR	2.25	R	46.3
15	WL440HQ	America	1.0 (0–1)	30.0	MR	2.50	R	45.1
16	Jiuquan	China	1.0 (0–1)	12.5	R	3.40	MR	42.8
17	5020	Canada	1.0 (0–2)	35.0	MR	2.50	R	41.2
18	Aohan	China	1.0 (1–3)	31.4	MR	3.86	SR	38.6
19	Super Nova	Canada	1.0 (0–2)	32.0	MR	3.80	SR	35.4
20	2295	Canada	2.0 (1–3)	46.7	S	4.00	SR	21.8
21	WL168HQ	America	2.0 (1–3)	52.0	S	3.40	MR	23.3
22	Instict	America	2.0 (1–3)	48.0	S	3.40	MR	18.3
23	Catera	France	2.0 (1–3)	45.7	S	4.14	S	19.2
24	Nongjing No. 1	China	3.0 (1–4)	60.0	S	4.20	S	8.9

Galling Index presented as median (Q1–Q3) based on 0–5 ordinal scale (n = 4 replicates per variety). Data analyzed using Kruskal–Wallis test (H = 156.3, df = 23, *p* < 0.001, ε^2^ = 0.72). Egg Mass Index calculated as average EMI score per variety based on 0–5 scale: 0 = no egg masses; 1 = 1–2; 2 = 3–10; 3 = 11–30; 4 = 31–100; 5 = >100 egg masses per plant. Composite Score (0–100 scale) integrates standardized galling index, egg mass count, and egg number, with higher values indicating greater resistance. Score = 50 + 10 × (mean of inverted z-scores). DI: HR = Highly Resistant (0–10), R = Resistant (11–20), MR = Moderately Resistant (21–40), S = Susceptible (41–60) l. EMI: R = Resistant (1.1–3.0), MR = Moderately Resistant (3.1–3.5), SR = Slightly Resistant (3.6–4.0), S = Susceptible (4.1–5.0).

## Data Availability

The original contributions presented in this study are included in the article. Further inquiries can be directed to the corresponding author.

## References

[1] Zhang Y., Wang L. (2025). Advances in basic biology of alfalfa (*Medicago sativa* L.): A comprehensive overview. Hortic. Res..

[2] Bouton J. (2012). An overview of the role of lucerne (*Medicago sativa* L.) in pastoral agriculture. Crop Pasture Sci..

[3] Shi S., Nan L., Smith K.F. (2017). The current status, problems, and prospects of alfalfa (*Medicago sativa* L.) breeding in China. Agronomy.

[4] Khan M.R. (2023). Nematode pests of agricultural crops, a global overview. Novel Biological and Biotechnological Applications in Plant Nematode Management.

[5] Tapia-Vázquez I., Montoya-Martínez A.C., De los Santos-Villalobos S., Ek-Ramos M.J., Montesinos-Matías R., Martínez-Anaya C. (2022). Root-knot nematodes (*Meloidogyne* spp.) a threat to agriculture in Mexico: Biology, current control strategies, and perspectives. World J. Microbiol. Biotechnol..

[6] Azlay L., El Boukhari M.E.M., Mayad E.H., Barakate M. (2023). Biological management of root-knot nematodes (*Meloidogyne* spp.): A review. Org. Agric..

[7] Walia R.K., Khan M.R. (2023). Root-knot nematodes (*Meloidogyne* spp.). Root-Galling Disease of Vegetable Plants.

[8] Vilela R.M.I.F., Kuster V.C., Magalhães T.A., Martini V.C., Oliveira R.M., De Oliveira D.C. (2023). Galls induced by a root-knot nematode in *Petroselinum crispum* (Mill.): Impacts on host development, histology, and cell wall dynamics. Protoplasma.

[9] Noureddine Y. (2021). Characterization of the Post-Transcriptional Regulations Involved in the Formation of Giant Cells Induced by Root-Knot Nematode. Ph.D. Thesis.

[10] Marquez J., Hajihassani A. (2023). Identification, diversity, and distribution of *Meloidogyne* spp. in vegetable fields of South Georgia, USA. Phytopathology^®^.

[11] Khan M.R., Rizvi T.F., Ansari M.S.A. (2023). Nematode problems in vegetables and ornamentals under protected cultivation and their sustainable management. Nematode Diseases of Crops and Their Sustainable Management.

[12] Castagnone-Sereno P., Danchin E.G., Perfus-Barbeoch L., Abad P. (2013). Diversity and evolution of root-knot nematodes, genus *Meloidogyne*: New insights from the genomic era. Annu. Rev. Phytopathol..

[13] Kavitha T., Prajwal B., Sunitha T., Prema G., Kranti K., Suneetha C., Priya K.T. (2025). *Meloidogyne*. Compendium of Phytopathogenic Microbes in Agro-Ecology.

[14] Leath K.T., Erwin D.C., Griffin G.D. (1988). Diseases and nematodes. Alfalfa Alfalfa Improv..

[15] Reddy P.P. (2021). Nematode Diseases of Crops and Their Management.

[16] Bui H.X., Desaeger J.A. (2021). Host suitability of summer cover crops to *Meloidogyne arenaria*, *M. enterolobii*, *M. incognita* and *M. javanica*. Nematology.

[17] Dobosz R., Krawczyk R. (2021). Precrop Effect of Red Clover (*Trifolium pratense* L.) and Alfalfa (*Medicago sativa* L.) on the Population Development of the Northern Root-Knot Nematode *Meloidogyne hapla* Chitwood, 1949 and on Succeeding Crops—A Pot Study. Agronomy.

[18] Abd-Elgawad M.M. (2024). Upgrading strategies for managing nematode pests on profitable crops. Plants.

[19] Putnam D.H. (2021). Factors influencing yield and quality in alfalfa. The Alfalfa Genome.

[20] Smiley R.W. (2021). Root-lesion nematodes affecting dryland cereals in the semiarid Pacific Northwest USA. Plant Dis..

[21] Elgin J., Welty R., Gilchrist D. (1988). Breeding for disease and nematode resistance. Alfalfa Alfalfa Improv..

[22] Yadav H., Roberts P.A., Lopez-Arredondo D. (2025). Combating Root-Knot nematodes (*Meloidogyne* spp.): From molecular mechanisms to resistant crops. Plants.

[23] Li X., Xing X., Tian P., Zhang M., Huo Z., Zhao K., Liu C., Duan D., He W., Yang T. (2018). Comparative transcriptome profiling reveals defense-related genes against *Meloidogyne incognita* invasion in tobacco. Molecules.

[24] Li X., Sun Y., Yang Y., Yang X., Xue W., Wu M., Chen P., Weng Y., Chen S. (2021). Transcriptomic and histological analysis of the response of susceptible and resistant cucumber to *Meloidogyne incognita* infection revealing complex resistance via multiple signaling pathways. Front. Plant Sci..

[25] Jinling L., Han J., Longhua S., Xianqi H., Zhirong Z., Xinrong W. (2003). Identification of species and race of root-knot nematodes on crops in southern China. Hua Zhong Nong Ye Da Xue Xue Bao J. Huazhong (Cent. China) Agric. Univ..

[26] Li C., Hu Y., Wang C. (2016). Identification of species and races of root-knot nematodes in greenhouse from Daqing city in Heilongjiang Province. Soils Crops.

[27] Hussey R., Barker K. (1973). A comparison of methods of collecting inocula of *Meloidogyne* spp., including a new technique. Plant Dis. Report..

[28] Taylor A., Sasser J. (1978). Biology, Identification and Control of Root-Knot Nematodes (Meloidogyne Species).

[29] Omwega C., Thomason I., Roberts P. (1988). A nondestructive technique for screening bean germ plasm for resistance to *Meloidogyne incognita*. Plant Disease.

[30] Yang B., Zhao Y., Guo Z. (2022). Research progress and prospect of alfalfa resistance to pathogens and pests. Plants.

[31] Thomas P. (2022). Game of’Mones: Comprehending Bemisia tabaci MEAM1 Nymph-Based Resistance and Defense Phytohormone Signaling in Alfalfa.

[32] Benjamin G. (2024). Analysis of the Tripartite Interaction Between Legumes, Symbiotic Nitrogen Fixing Bacteria and Aphids. Ph.D. Thesis.

[33] Mishra P., Saini P., Patni V. (2024). Biochemical dynamics during development of insect-induced plant galls: A review. J. Plant Dis. Prot..

[34] Petrikovszki R., Cseresnyés I., Bárányos F., Molnár A.G., Boros G. (2023). Early detection of root-knot nematode (*Meloidogyne incognita*) infection by monitoring root dielectric response non-destructively. Int. Agrophys..

[35] Bernard G.C., Khan M.R. (2025). Understanding the Mechanisms of Nematode Disease Complexes. Nematode Disease Complexes in Agricultural Crops.

[36] Jia Y., Runnan Z., Yu Y., Lamlom S.F., Hu Y., Li J., Li H., Wang J. (2025). A Novel Mechanism Underlying Resistance to Soybean Cyst Nematode in the Resistant Soybean HN531. Agronomy.

[37] Rubiales D., Barilli E., Rispail N. (2023). Breeding for biotic stress resistance in pea. Agriculture.

